# Supplementary material for: Single-cell and spatial analysis reveal interaction of FAP+ fibroblasts and SPP1+ macrophages in colorectal cancer
Source: Nat Commun. 2022 Apr 1;13:1742. doi: 10.1038/s41467-022-29366-6 (PMC8976074; doi:10.1038/s41467-022-29366-6)
Supplement: Supplementary file 1 — Supplementary Information [file 41467_2022_29366_MOESM1_ESM.pdf]

Supplementary Information for:

Single-cell and spatial analysis reveal interaction of *FAP*⁺ fibroblasts and *SPP1*⁺ macrophages in colorectal cancer

Jingjing Qi[1,2,3], Hongxiang Sun[1,2], Yao Zhang[4], Zhengting Wang[4], Zhenzhen Xun[1], Ziyi Li[1], Xinyu Ding[1], Rujuan Bao[1], Liwen Hong[4], Wenqing Jia[1], Fei Fang[1], Hongzhi Liu[1,2], Lei Chen[1], Jie Zhong[4], Duowu Zou[4], Lianxin Liu[5], Leng Han[6], Florent Ginhoux[1,7], Yingbin Liu[3], Youqiong Ye[1,2,8] & Bing Su[1,2,8]

[1]Shanghai Institute of Immunology, Department of Immunology and Microbiology, and the Ministry of Education Key Laboratory of Cell Death and Differentiation, Shanghai Jiao Tong University School of Medicine, Shanghai, China

[2]Shanghai Jiao Tong University School of Medicine-Yale Institute for Immune Metabolism, Shanghai Jiao Tong University School of Medicine, Shanghai, China

[3]Department of Biliary and Pancreatic Surgery, Renji Hospital, Shanghai Jiao Tong University School of Medicine, Shanghai, China

[4]Department of Gastroenterology, Ruijin Hospital, Shanghai Jiao Tong University School of Medicine, Shanghai, China

[5]Department of Hepatobiliary Surgery, Anhui Province Key Laboratory of Hepatopancreatobiliary Surgery, The First Affiliated Hospital of USTC, Division of Life Sciences and Medicine, University of Science and Technology of China, Hefei 230001, China.

[6]Department of Biochemistry and Molecular Biology, The University of Texas Health Science Center at Houston McGovern Medical School, Houston, TX 77030, USA

[7]Singapore Immunology Network (SIgN), A*STAR, 8A Biomedical Grove, Immunos Building, Level 3 and 4, Singapore 138648, Singapore

[8]These authors jointly supervised this work: Youqiong Ye, Bing Su. Email: youqiong.ye@shsmu.edu.cn; bingsu@sjtu.edu.cn.

This PDF file includes:

Supplementary Figure 1-8

Supplementary Table 1-2

Supplementary Note 1-4

Supplementary References

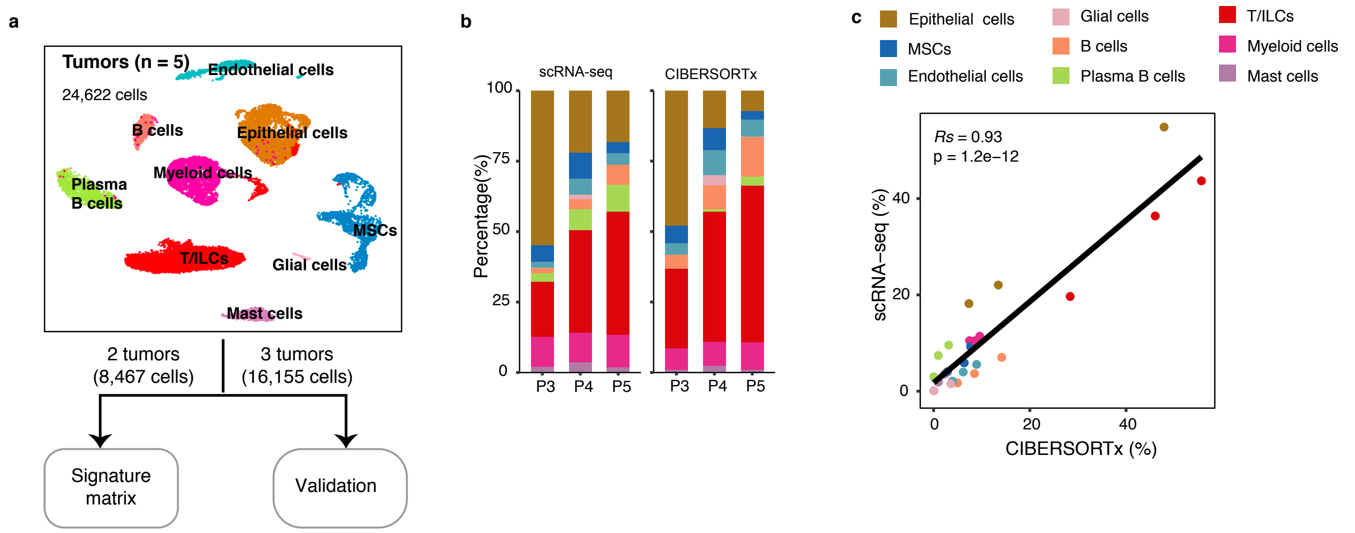

Supplementary Fig.1 Robustness assessment of CIBERSORTx. (a) UMAP visualization of our scRNA-seq data from 5 CRC tissues (upper) and approach for testing single-cell deconvolution performance (bottom). **(b-c)** Concordance between cell type proportions of nine major cell types measured by CIBERSORTx deconvolution and scRNA-seq for 3 held-out CRC tissues reconstructed from single-cell transcriptomic data, related to **(b)**. Shown are stacked bar plots **(b)** and Pearson correlations **(c)** of observed versus expected cell subset proportions. Source data are provided as a Source Data Supplementary Figure 1a-c.

a

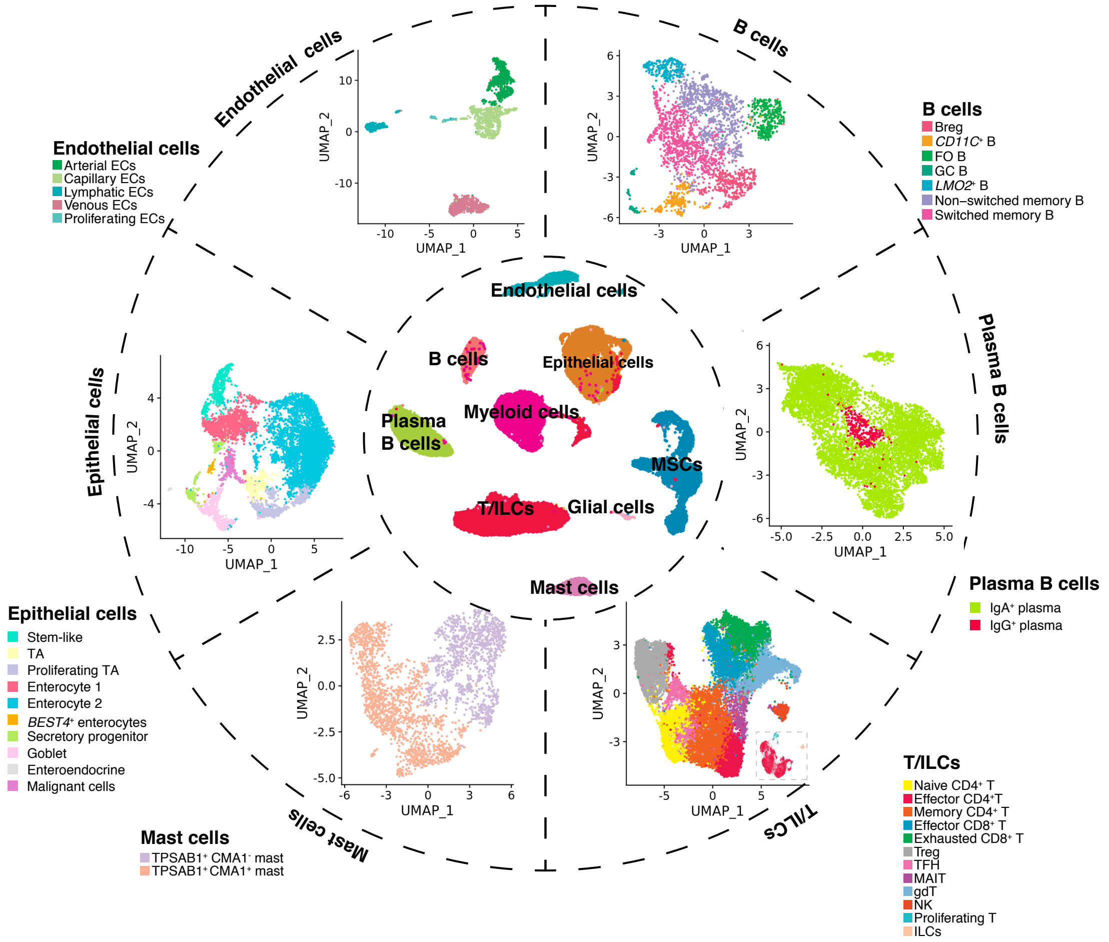

b

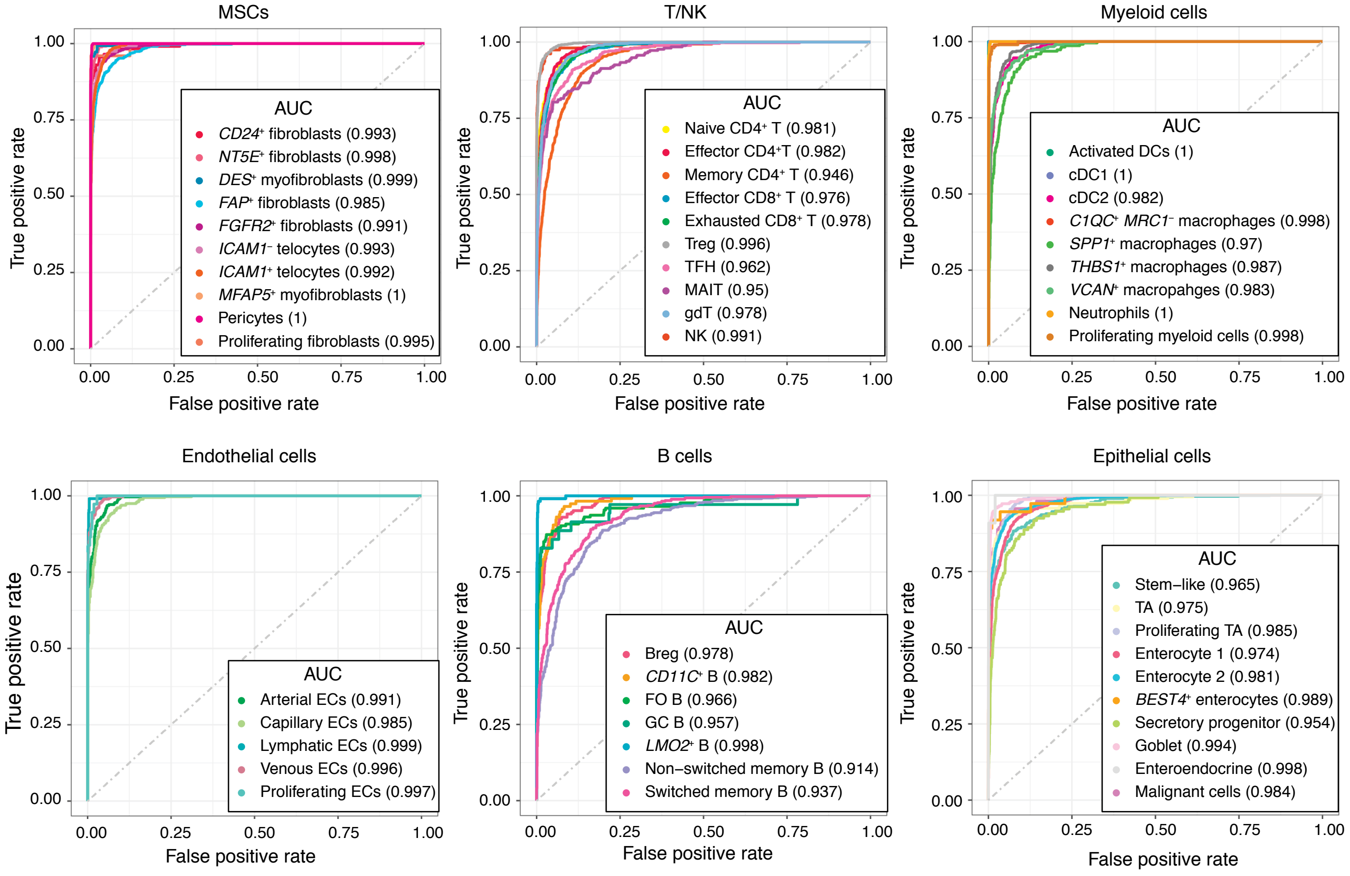

(*To be continued*)

c

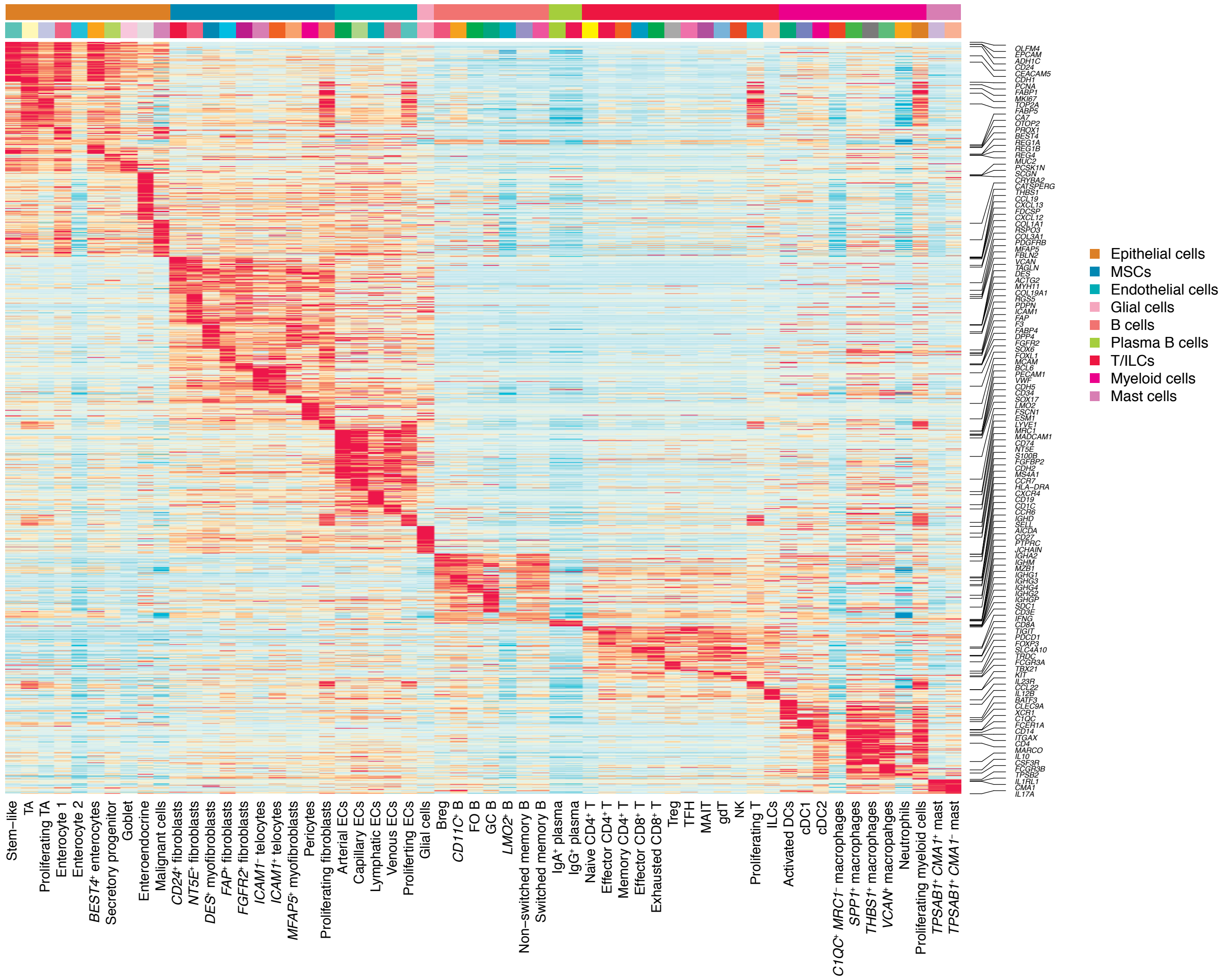

d

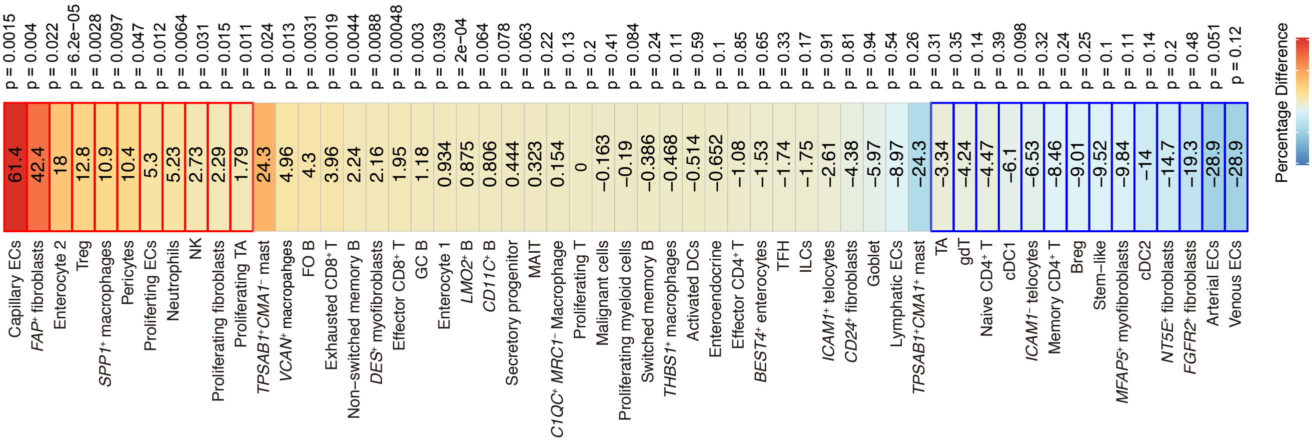

(*To be continued*)

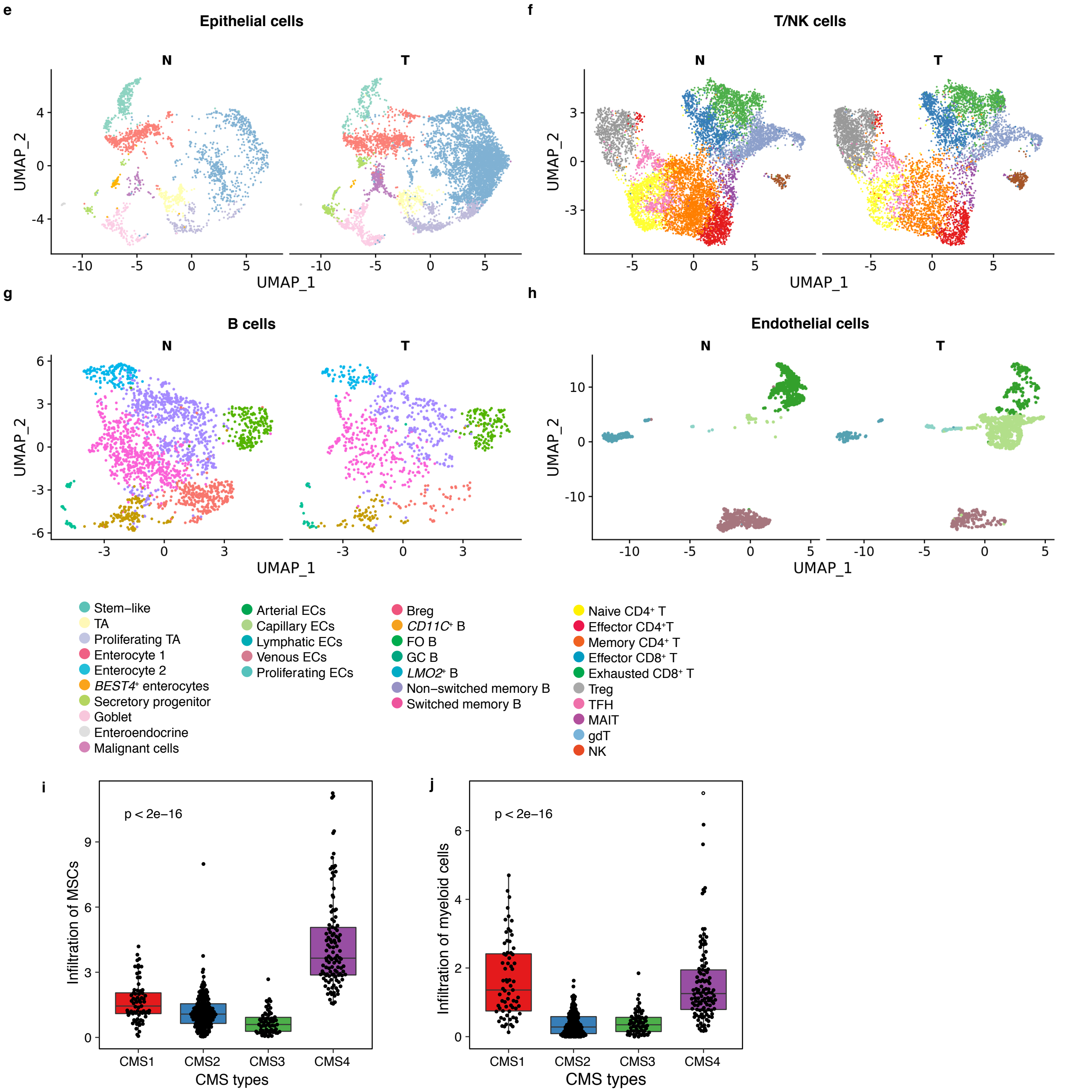

Supplementary Fig.2 Characterization and machine method validation of cell subtypes atlas. (a) UMAP of main subtypes (inner circle), and subtypes of each global cell types (outer circle) recovered from normal mucosa and tumor tissue from CRC patients. Each dot represents one cell. Cells are color-coded by cell type accordingly. **(b)** Cell subtypes reproducibility was assessed by machine learning methods. For all subtypes, false positive rate against true positive rate is shown based on the probability thresholds of the logistic regression. 50% chance of false classification is shown by dash lines. AUC, area under the curve. **(c)** Expression of subtype-specific markers genes (rows) across each cell subtypes (columns). Gene names are shown on the right and subtype names are shown on the bottom. **(d)** The difference in the proportion of each cell type in each major type between tumor tissue and normal mucosa. Numbers in the squares represent differences of cell percentages. Red square, the proportion of cell type is upregulated in tumor tissue compared to normal mucosa; blue square, the proportion of cell type is downregulated. P-values were calculated by paired two-sided Student's t-test and were shown above each square. **(e-h)** UMAP of the composition of epithelial cells **(e)**, T cells **(f)**, B cells **(g)**, and endothelial cells **(h)** subtypes in adjacent and tumor tissues. **(i-j)**The infiltration of MSCs **(i)** and myeloid cells **(j)** in different CMS types, including CMS1 (n = 69), CMS2 (n = 195), CMS3 (n = 62), and CMS4 (n = 114). Statistical analysis was calculated by ANOVA test. The boxes show the median ± 1 quartile, with the whiskers extending from the hinge to the smallest or largest value within 1.5 × the IQR from the box boundaries. Source data are provided as a Source Data Supplementary Figure 2a-b, e-j.

a

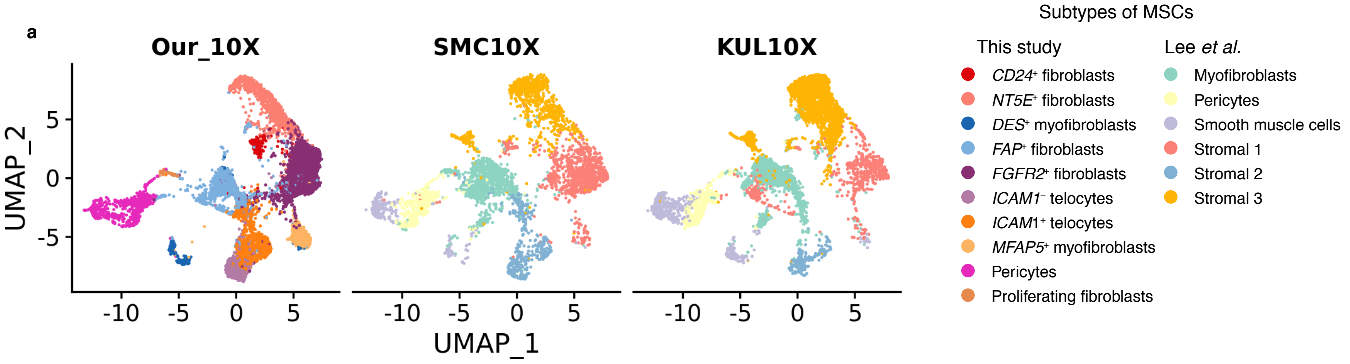

b

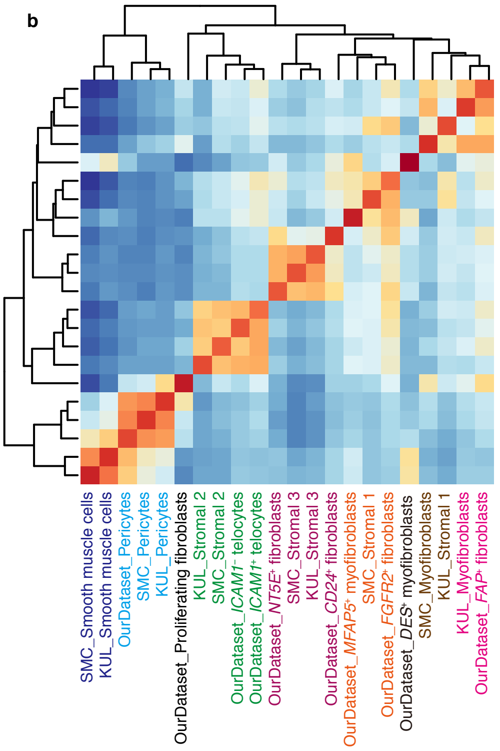

c

In this study	Lee *et al.*
CD24⁺ fibroblasts	Stromal 3
NT5E⁺ fibroblasts	Stromal 3
FAP⁺ fibroblasts	Myofibroblasts
FGFR2⁺ fibroblasts	Stromal 1
Proliferating fibroblasts	-
DES⁺ myofibroblasts	Smooth muscle cells
MFAP5⁺ myofibroblasts	Stromal 1
Pericytes	Pericytes/Smooth muscle cells
ICAM1⁻ telocytes	Stromal 2
ICAM1⁺ telocytes	Stromal 2

d

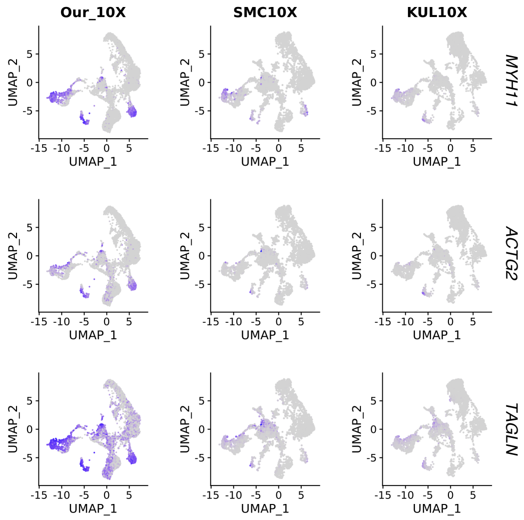

Supplementary Fig. 3 Integration analysis of stromal cells with published datasets, related to Fig. 3. (a) UMAP of the stromal cells obtained from this study and Lee *et al.*'s dataset. **(b)** Unsupervised hierarchical clustering of Spearman correlation among CRC stomal cell subsets based on the cell annotation in this study and Lee *et al*. **(c)** Table comparing the stomal cell subsets between this study and Lee *et al*. **(d)** Feature plots showing relative expression of *MYH11*, *ACTG2*, and *TAGLN* in each of stromal subsets on UMAP space as in **(a)**. Source data are provided as Source Data Supplementary Figure 3a-b, d.

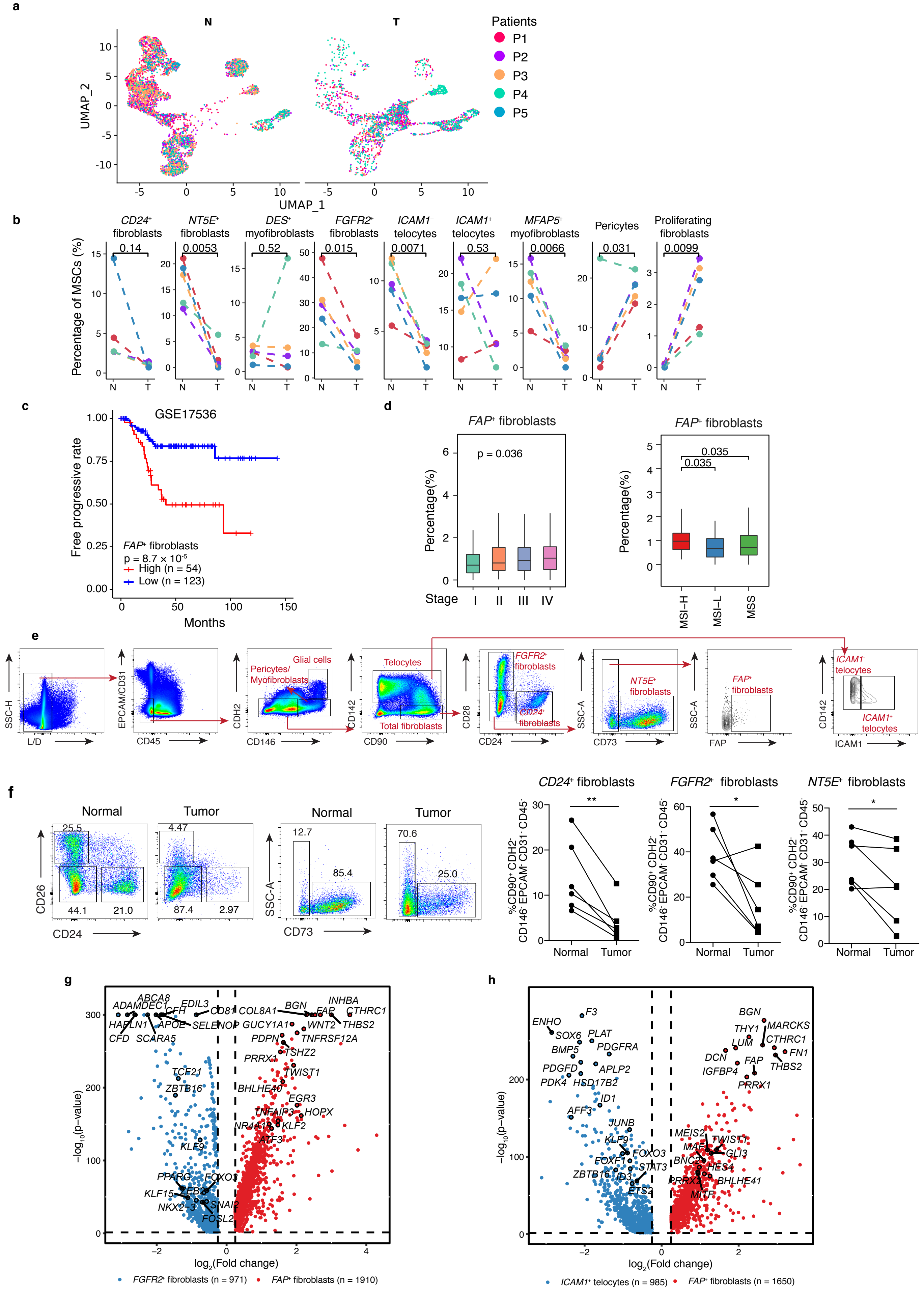

(*To be continued*)

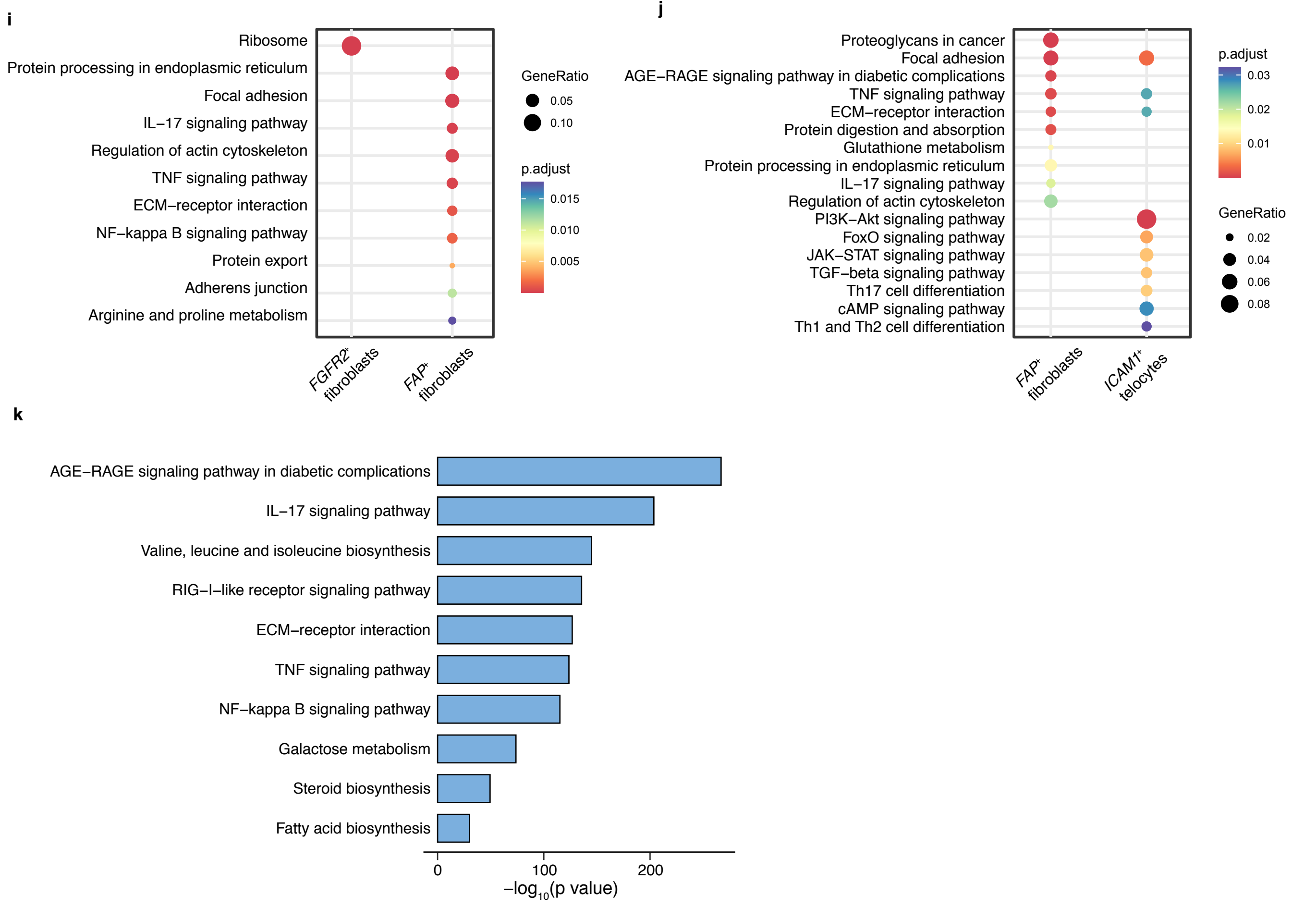

Supplementary Fig.4 Characterization of mesenchymal stromal cells, related to Fig. 3. (a) UMAP visualization of MSCs subtypes colored by donor origin. **(b)** Comparation of percentages of MSCs subtypes in indicated tissues. Statistical analysis was calculated by two-sided paired Student's t-test. Donors are color-coded as in **(a)**. Each group has 5 samples. **(c)** Kaplan-Meier progression free survival curves grouped by *FAP*$^+$ fibroblasts infiltration in GSE17536. **(d)** Infiltration of *FAP*$^+$ fibroblasts among tumor stage (left), including Stage I (n = 57), II (n = 104), III (n = 71), IV (n = 43) and MSI status (right), including MSI-H (n = 40), MSI-L (n = 45), MSS (n = 190) in TCGA COAD patients. The boxes show the median ± 1 quartile, with the whiskers extending from the hinge to the smallest or largest value within 1.5 × the IQR from the box boundaries. **(e)** Gating strategy to analyze MSCs subtypes. Total fibroblasts (live CD45$^-$ EPCAM$^-$ CD45$^-$ CDH2$^-$ CD146$^-$ CD142$^-$ CD90$^+$); *CD24*$^+$ fibroblasts (CD26$^-$ CD24$^+$); *FGFR2*$^+$ fibroblasts (CD24$^-$ CD26$^+$); *NT5E*$^+$ fibroblasts (CD24$^-$ CD26$^-$ CD73$^+$) and *FAP*$^+$ fibroblasts (CD24$^-$ CD26$^-$ CD73$^-$ FAP$^+$). Moreover, glial cells (live CD45$^-$ EPCAM$^-$ CD45$^-$ CDH2$^+$ CD146$^+$); pericytes and myofibroblasts (live CD45$^-$ EPCAM$^-$ CD45$^-$ CDH2$^-$ CD146$^+$). Telocytes were gated on live CD45$^-$ EPCAM$^-$ CD45$^-$ CDH2$^-$ CD146$^-$ CD142$^+$ cells, and further gated based on ICAM1 expression. **(f)** FACS plots (left) and dot plots (right) show the comparison of *CD24*$^+$ fibroblasts, *CD26*$^+$ fibroblasts and *NT5E*$^+$ fibroblasts in normal mucosa (n=6) and tumor tissue(n=6). Fibroblasts are gated as in **(e)**. p= 0.0051, 0.0347, and 0.0476, respectively. **(g-h)** Volcano plots showing differentially expressed genes between *FGFR2*$^+$ fibroblasts and *FAP*$^+$ fibroblasts **(g)** or *ICAM1*$^+$ telocytes and *FAP*$^+$ fibroblasts **(h)**. x axis for log2-transformed fold changes and y axis for -log10-transformed p-value. Top 10 differential marker and transcription factor genes were highlighted. **(i-j)** KEGG pathway analysis of the differential expressed genes between *FGFR2*$^+$ fibroblasts and *FAP*$^+$ fibroblasts **(i)** or *ICAM1*$^+$ telocytes and *FAP*$^+$ fibroblasts **(j)**. Size of the bubbles show the number of genes enriched in each pathway whereas the color intensity represents the enrichment significance. **(k)** Bar plots showing the unique KEGG pathways that *FAP*$^+$ fibroblasts enriched compared with other stromal cells. N, normal mucosa; T, tumor tissue; CRC, colorectal cancer. MSI-H, microsatellite instability-high; MSI-L, microsatellite instability-low; MSS, microsatellite stability. A paired two-sided Student's t-test was used to assess the difference in **(b)** and **(f)**. One-way AVNOVA test in **(d)**. p-value calculated by two-sided Wilcox-test in **(g, h)**.Fisher's test in **(i, j, k)**, and adjusted p value by False Discovery Rate. $p < 0.05$ is considered as a statistically significant difference. *p<0.05, **p<0.01. Source data are provided as Source Data Supplementary Figure 4a-d, f-k.

a

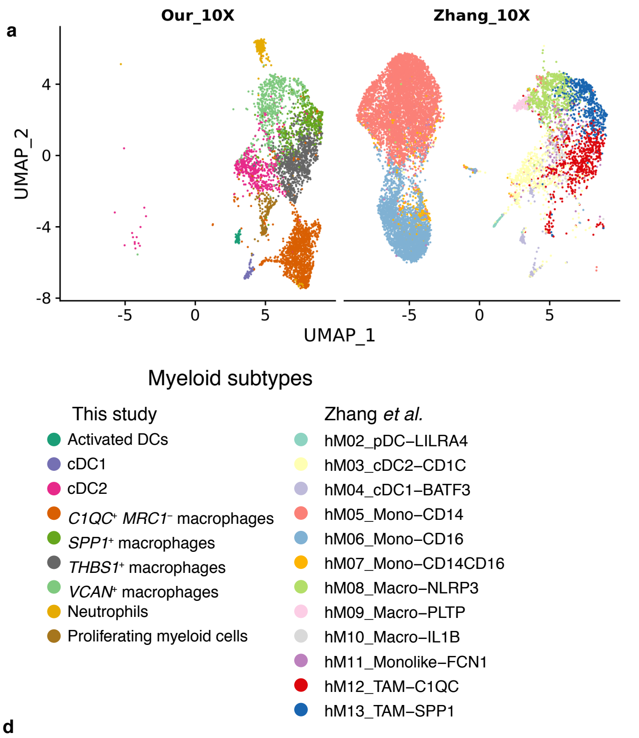

b

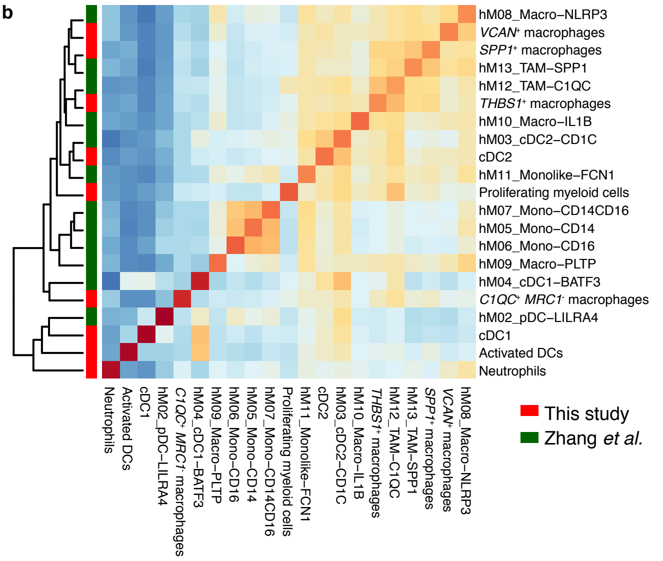

c

In this study	Zhang *et al.*
Activated DCs	cDC1-BATF3
cDC1	cDC1-BATF3
cDC2	cDC2-CD1C
C1QC+*MRC1*- macrophages	-
SPP1+ macrophages	TAM-SPP1
THBS1+ macrophages	TAM-C1QC
VCAN+ macrophages	Macro-NLRP3
Neutrophils	-
Proliferating myeloid cells	-
-	Mono-CD14, Mono-CD16, MonoCD14CD16, Monolike-FCN1
-	Macro–PLTP
-	Macro–IL1B
-	pDC-LILRA4

d

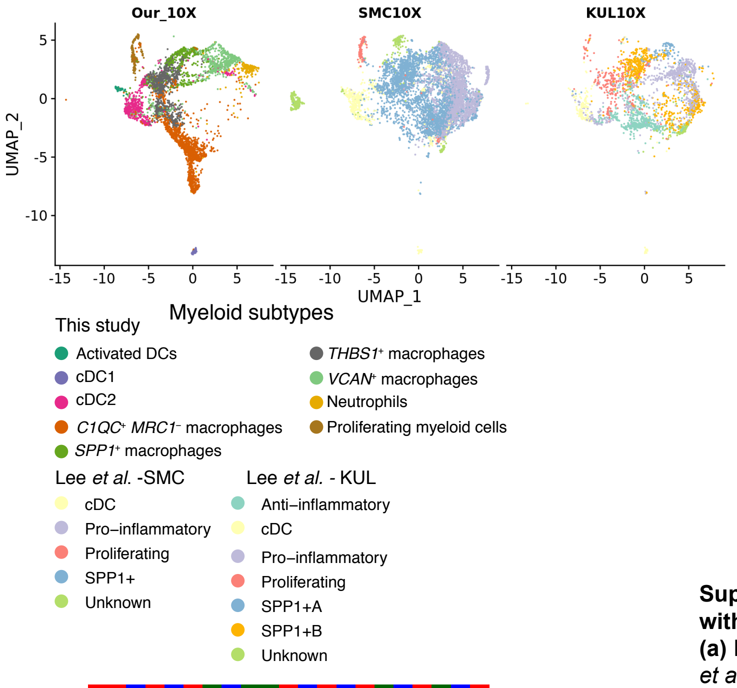

f

In this study	Lee *et al.* SMC	Lee *et al.* KUL
Activated DCs	-	-
cDC1	-	-
cDC2	cDC	cDC
C1QC+*MRC1*- macrophages		Anti-inflammatory
SPP1+ macrophages	SPP1+	SPP1+B
THBS1+ macrophages		Proliferating
VCAN+ macrophages	Pro–inflammatory	Pro–inflammatory
Neutrophils	-	-
Proliferating myeloid cells	Proliferating	-
-	Unknown	-
		SPP1+A
-	-	Unknown

e

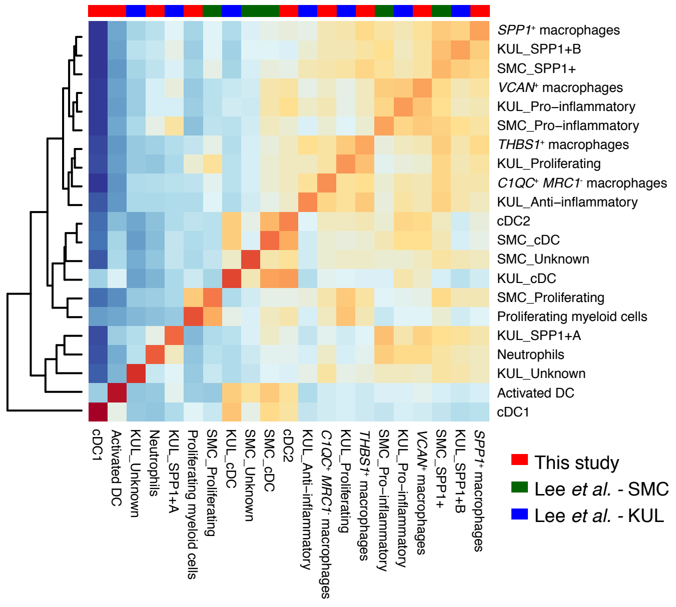

Supplementary Fig. 5 Integration analysis of myeloid cells with published datasets, related to Fig.4.
(a) Integration of myeloid cells between this study and Zhang *et al.*'s dataset presented in UMAP dimension. Zhang *et al.*'s dataset was merged with data from this study, batch effect corrected with Seurat Integration, and then projected onto the bidimensional UMAP space. **(b)** Unsupervised hierarchical clustering Spearman correlation among CRC myeloid cell subsets based on the cell annotation in this study and Zhang *et al.* **(c)** Table summarizes CRCmyeloid cell subsets between this study and Zhang *et al.* **(d)** Integration of myeloid cells between this study and Lee *et al.*'s dataset presented in UMAP dimension. Two cohorts (SMC, Samsung Medical Center; KUL, Katholieke Universiteit Leuven) of Lee *et al.*'s datasets were merged with data from this study, batch effected with Seurat Integration, and then projected onto the bidimensional UMAP space. **(e)** Unsupervised hierarchical clustering Spearman correlation among CRC myeloid cell subsets based on the cell annotation in this study and Lee *et al.* **(f)** Table summarizes the CRC myeloid cell subsets between this study and Lee *et al.* Source data are provided as Source Data Supplementary Figure 5a-b, d-e.

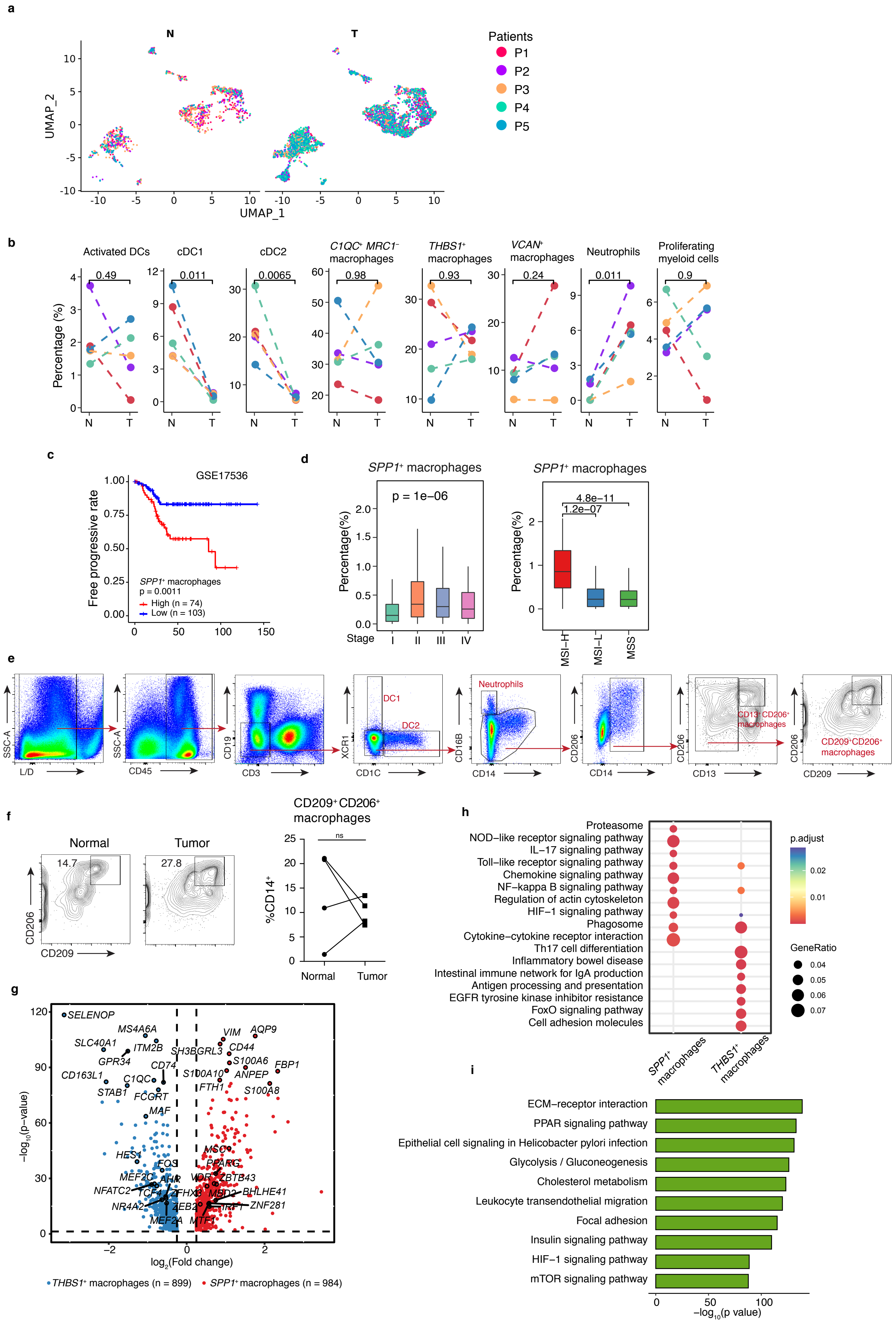

(*Legend on next page*)

Supplementary Fig.6 Characterization of myeloid cells, related to Fig. 4. (a) UMAP showing the even distribution of myeloid subtypes across patients in both normal mucosa and tumor tissue. Each cell was colored by donor. **(b)** Percentages of each myeloid subtypes were compared between normal mucosa and tumor tissue from scRNA-seq. Color-coded dots represent to donors in scRNA-seq. Normal mucosa (n = 5) and tumor tissue (n = 5). **(c)** The Kaplan-Meier curves show the progression free survival in colon cancer microarray GSE17536 grouped by gene signatures expression of *SPP1*$^+$ macrophages. **(d)** Distribution of *SPP1*$^+$ macrophages in tumor tissue of different stages (left), including Stage I (n = 57), II (n = 104), III (n = 71), IV (n = 43), and MSI status (right), including MSI-H (n = 40), MSI-L (n = 45), MSS (n = 190), of TCGA COAD. MSI-H, microsatellite instability-high; MSI-L, microsatellite instability-low; MSS, microsatellite stability. The boxes indicate the median ± 1 quartile, with whiskers extending from the hinge to the smallest or largest value within 1.5 interquartile range from the box boundaries. **(e)** Representative gating strategy of myeloid subtypes in human CRC tissue. Macrophages were gated by exclusion the expression of CD3 (T cell marker), CD19 (B cell marker), XCR1, CD1C (DC1 and DC2 respectively), and CD16B (neutrophils marker), and were CD45 positive and CD14 positive. *SPP1*$^+$ macrophages were both CD206 and CD13 positive. *THBS1*$^+$ macrophages were CD13 negative and CD209, CD206 positive. **(f)** Counter plots (left) and statistical analysis of *THBS1*$^+$ macrophages (CD209$^+$ CD206$^+$) percentages among CD14$^+$ cells (right) in normal mucosa (n=4) and tumor tissue (n=4) of CRC patients. ns, not significant. **(g)** Differential expressed genes between *THBS1*$^+$ macrophages and *SPP1*$^+$ macrophages. x-axis is log2-transformed fold change, y-axis is statistical significance which calculated by two-sided Wilcox-test. The black circle and label of gene name indicates top 10 differential markers or top 10 transcription factors. **(h)** Dot plots showing the enrichment of KEGG pathways of differential expressed genes between *THBS1*$^+$ macrophages and *SPP1*$^+$ macrophages. Dot size and color intensity represent genes enriched in each pathway and enrichment significance respectively. **(i)** Unique KEGG pathways of *SPP1*$^+$ macrophages compared with other myeloid cells. A paired two-sided Student's t-test was used to assess the difference in **(b)** and **(f)**. A two-sided log-rank test in (**c**). One-way ANOVA in **(d)**. Fisher's test in **(h-i)** and p value adjusted by FDR. $p < 0.05$ is considered as a statistically significant difference. Source data are provided as Source Data Supplementary Figure 6a-d, f-I.

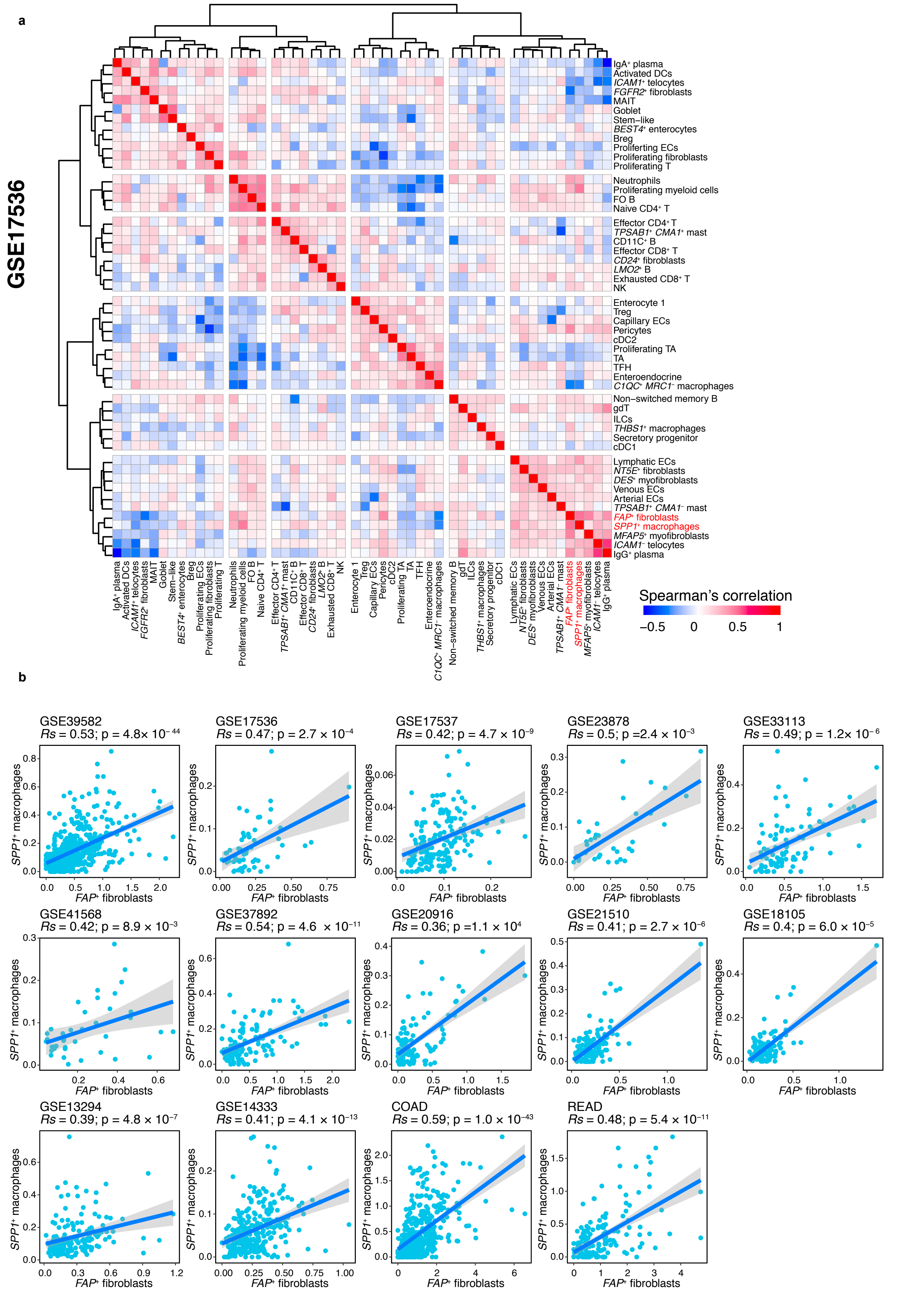

Supplementary Fig. 7 *FAP*⁺ fibroblasts and *SPP1*⁺ macrophages infiltration is highly correlated, related to Fig. 5. (a) Spearman correlation heatmap of 58 cell types identified by scRNA-seq in GSE17536 with *Rs* ranging from -0.5 to 1. **(b)** Scatter plots show the correlation between the infiltration of *FAP*⁺ fibroblasts and *SPP1*⁺ macrophages across 14 independent datasets with CRC, including GSE39582 (n = 566); GSE17536 (n = 177); GSE17537 (n = 55); GSE23878 (n = 35); GSE33113 (n = 90); GSE41568 (n = 133); GSE37892 (n = 130); GSE20916 (n = 111); GSE21510 (n = 123); GSE18105 (n = 94); GSE13294 (n = 155); GSE14333 (n = 290); TCGA COAD (n= 495), TCGA READ (n = 140). The error band indicates 95% confidence interval. Source data are provided as Source Data Supplementary Figure 7a-h.

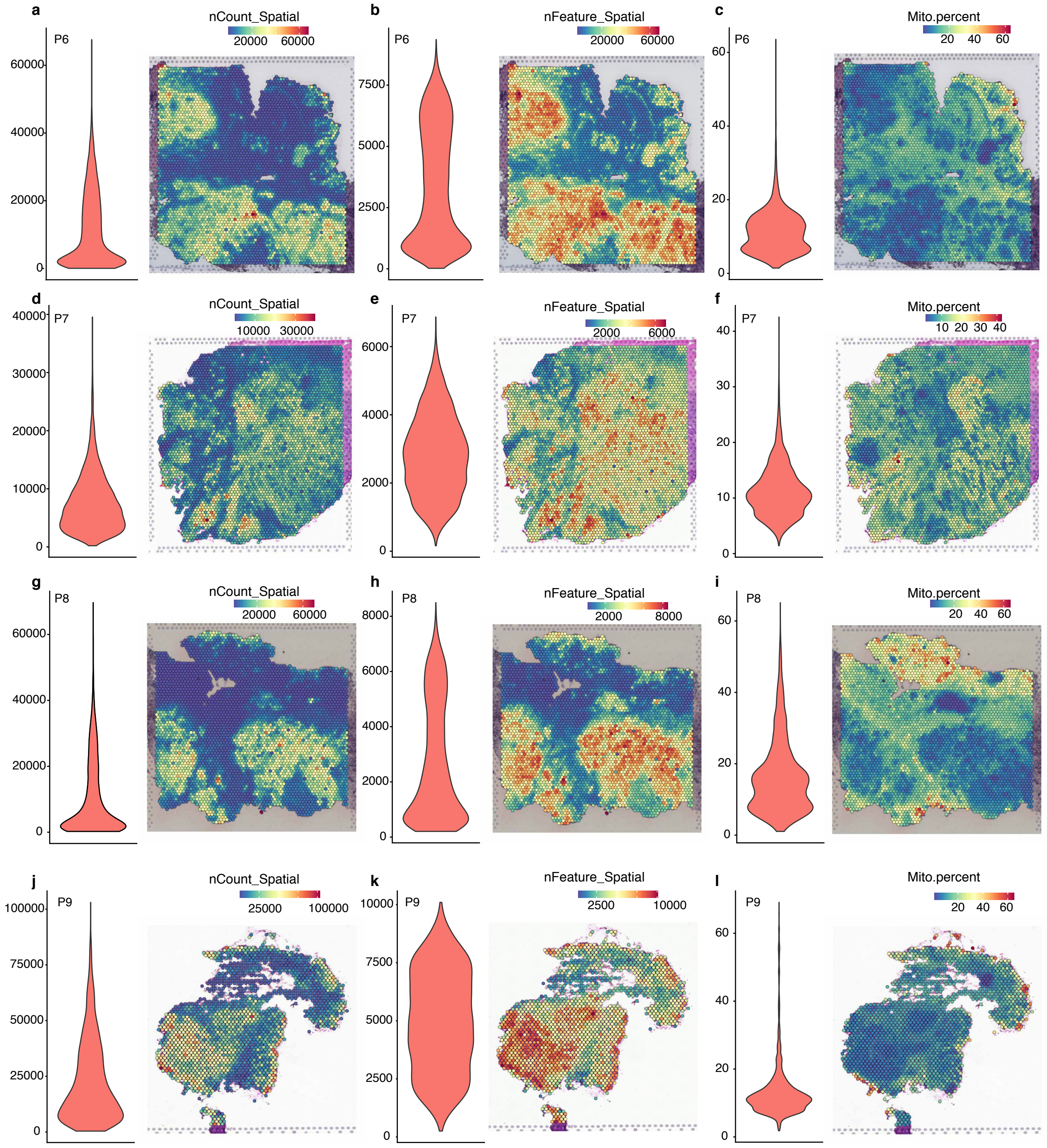

Supplementary Fig.8 Features of spatial transcriptomics. The numbers of UMI counts **(a, d, g, j)**, gene features **(b, e, h, k)**, and percentages of mitochondrial genes **(c, f, i, l)** in patient #6 **(a-c)**, patient #7 **(d-f)**, patient #8 **(g-i)**, and patient #9 **(j-l)**. Source data are provided as Source Data Supplementary Figure 8a-l.

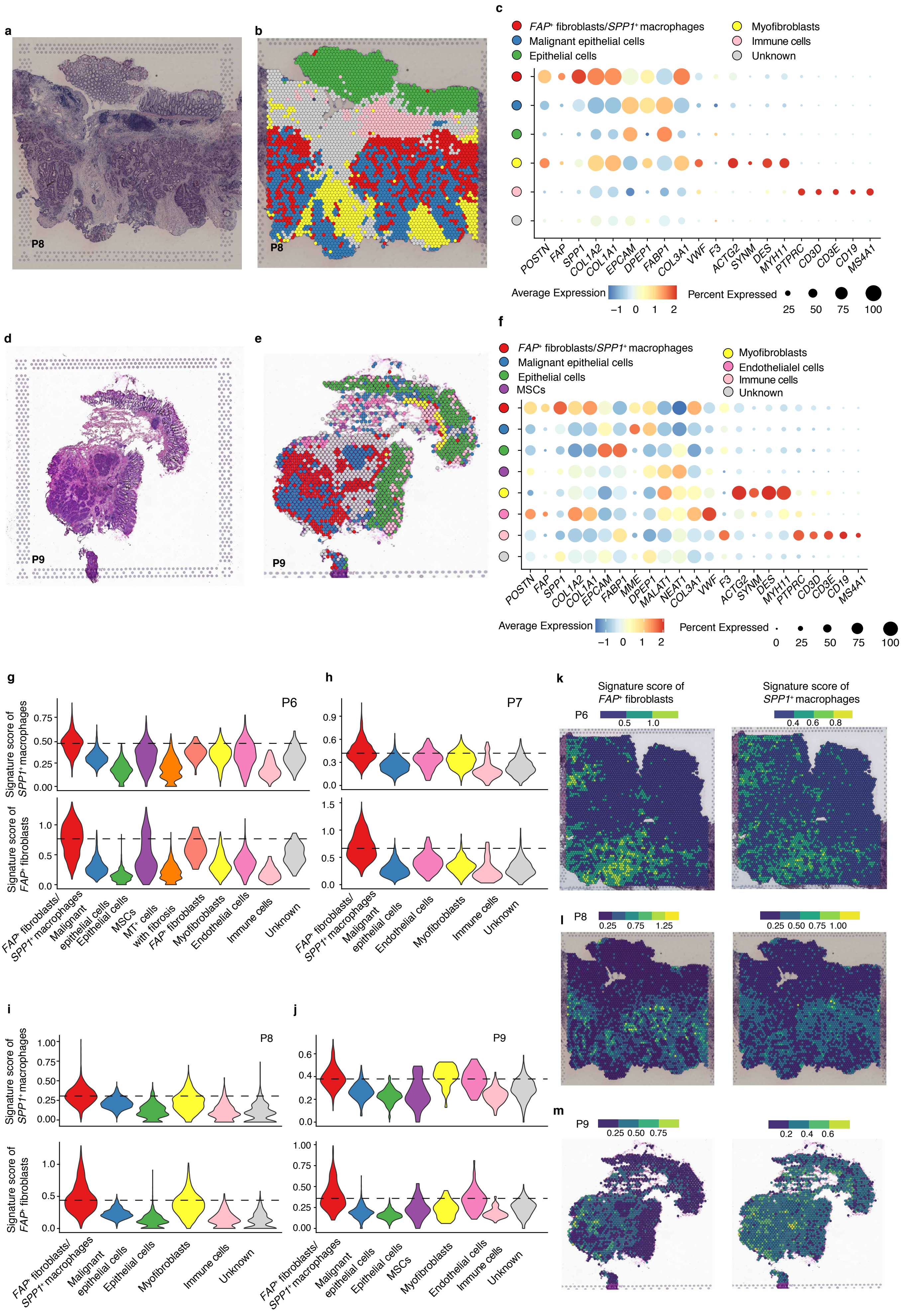

(*To be continued*)

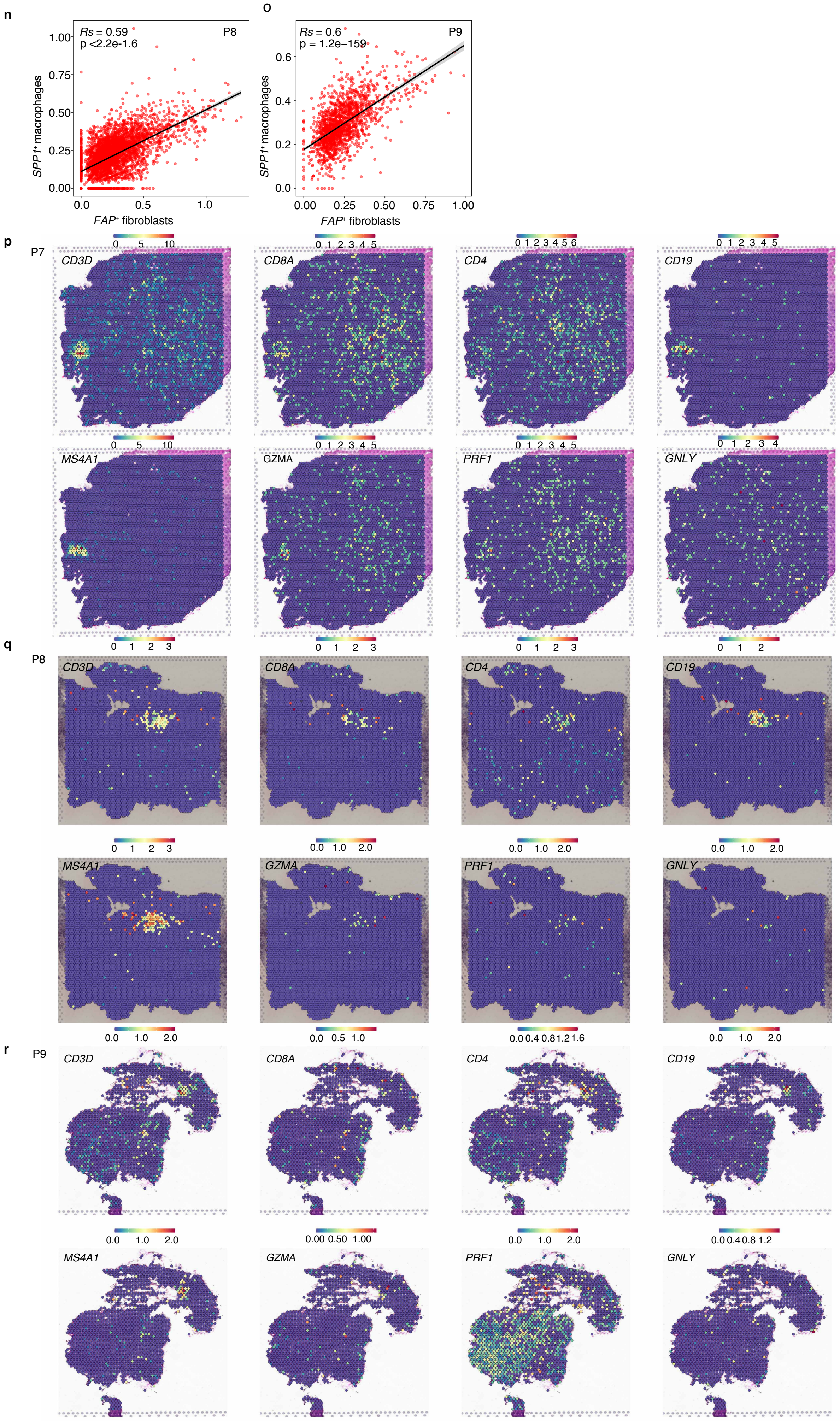

(Legend on next page)

Supplementary Fig. 9 Close localization of *FAP*⁺ fibroblasts and *SPP1*⁺ macrophages revealed by spatial transcriptomics, related to Fig. 6. (a-f) HE staining, unbiased clustering, and known markers expression for each cluster for CRC patient #8 **(a-c)**, and patient #9 **(d-f)**, respectively. H&E staining **(a, d)** was repeated twice for one tumor block. **(g-j)** Violin plots of signature score of *SPP1*⁺ macrophages (upper) and *FAP*⁺ fibroblasts (bottom) of individual spots derived from scRNA-seq data for each cluster in patient #6 **(g)**, #7 **(h)**, #8 **(i)** and #9 **(j)**, respectively. Dotted boxes outline clusters with highest of signature score, and the dashed line indicates the median of signature score. **(k-m)** Spatial feature plots of signature score of *FAP*⁺ fibroblasts (left) and *SPP1*⁺ macrophages (right) in tissue sections in patient # 6 **(k)**, #8 **(l)**, and #9 **(m)**. **(n-p)** Spatial feature plots of signature score of epithelial cells in patient #6 **(n)**, #8 **(o)**, and #9 **(p)**. **(q-r)** The Pearson correlation of signature score of *FAP*⁺ fibroblasts (x-axis) and *SPP1*⁺ macrophages (y-axis) in *FAP*⁺ fibroblasts/*SPP1*⁺ macrophages cluster in patient #8 **(q)** and patient #9 **(r)**. **(s)** Spatial feature plots showing expression of *CD3D*, *CD8A*, *CD4*, *CD19*, *MS4A1*, *GZMA*, *PRF1*, and *GNLY* in tissue sections of patient #7, #8, and #9. Source data are provided as Source Data Supplementary Figure 9b-c, e-m, q-t, u.

Supplementary Tables

Supplementary Table 1 Clinical characteristics of CRC patients in this study

Characteristics	P1	P2	P3	P4	P5
**Age**	30-40	70-80	70-80	50-60	50-60
**Gender**	Female	Male	Male	Male	Female
**Anatomical region**	Colon	Colon	Rectum	Rectum	Rectum
**pTNM: T**	3	3	2	4a	3
**pTNM: N**	2a	0	0	2a	0
**pTNM: M**	0	0	0	0	0
**Stage**	IIIb	IIa	I	IIIc	IIa
**Tumour size**	5×4×2 cm	4.5×3×1.5 cm	3×3×1.5 cm	5×4×1.5 cm	3.5×3.5×1.5 cm
**Grade (Differntiated)**	Low	Moderately	Low or moderately	Moderately	Moderately

Supplementary Table 2 The information of 14 independent CRC cohort from TCGA and GEO, including the accession number, platform of microarray, the number of tumor and normal samples, clinical characteristics (stage, gender).

Accession number	Platform	Tumor samples	Normal samples	Survival	Stage	Gender
TCGA-COAD	Illumina	459	41	OS/PFS	I: 74; II: 172; III: 126; IV: 61	F:216; M:243
TCGA-READ	Illumina	170	10	OS/PFS	I: 33; II: 52; III: 54; IV: 27	F:78; M:92
GSE13294	GPL570	155	0	NA	NA	NA
GSE14333	GPL570	290	0	PFS	A:44;B:95;C:93;D:61	F:89;M:99;NA:105
GSE17536	GPL570	177	0	OS/PFS	I:24;II:57;III:57;IV:39)	M:96;F:81
GSE17537	GPL570	55	0	OS/PFS	I:4;II:15;II:19;IV:17	M:29;F:26
GSE18105	GPL570	94	17	NA	II/III	NA
GSE20916	GPL570	111	34	NA	NA	M:51;F:94
GSE21510	GPL570	123	25	NA	I:13;II:37;III:34;IV:20	NA
GSE23878	GPL570	35	24	NA	NA	F:19;M:40
GSE33113	GPL570	90	6	NA	stage II:90	M:42;F:48;NA:6
GSE37892	GPL570	130	0	NA	II:73; III:57	M:69;F:61
GSE39582	GPL570	566	19	OS	I:37; II:264; III:205; IV4:60	M:310;F:256
GSE41568	GPL570	133	0	NA	NA	NA

TCGA, The Cancer Genomic Atlas; COAD, Colon Adenocarcinoma; READ, Rectum Adenocarcinoma; OS, overall survival; PFS, progressive free survival; F, female; M, Male.

Supplementary Note 1 | Validation of CIBERSORTx performance

CIBERSORTx is extended from CIBERSORT, further enables the building of custom signature matrixces from single-cell or flow-sorted bulk transcriptomic data and reconstruction of cell-specific transcriptional profiles[1]. We compared concordance between cell type proportions measured by CIBERSORTx deconvolution and scRNA-seq for held-out tumors reconstructed from our single-cell data to evaluate the performance of CIBERSORTx, based on the validation method provided by the developers of CIBERSORTx. Our dataset has five samples, we created a signature matrix from a training cohort consisting of two CRC specimens, used the remaining three CRC specimens as the test dataset (Supplementary Fig. 1a). As Figure 1b shown, the signature matrix distinguished epithelial cells, MSCs, endothelial cells, glial cells, B cells, plasma cells, T/ILCs, myeloid cells, and mast cells. When evaluated using reconstructed tumor samples, deconvolution results were highly concordant with ground truth cell proportions (Supplementary Fig. 1b), scRNAseq and CIBERSORTx proportions for 9 cell types in 3 held-out CRC specimens showed significantly high correlation ($Rs = 0.93$, $p = 12 \times 10^{-12}$; Supplementary Fig. 1c).

Supplementary Note 2 | Alteration of cell infiltration between adjacent normal and tumor tissue support the remodeling of tumor microenvironment in CRC

To identify subclusters within each of the major cell lineages defined above, we performed cluster analysis within each cell lineage (Supplementary Fig. 2a). The robustness and reproducibility of subclusters in each cell lineage was validated by a machine-learning approach based on the random forest algorithm (Supplementary Fig. 2b; see Methods). Since adjacent normal tissues (but not tumor samples) were treated with EDTA, the following analysis excluded epithelial cell populations (Supplementary Fig. 2d and 2e). T cells and innate lymphoid cells (such as natural killer cells) are the key cell types responsible for the depletion of tumor cells and tumor immunomodulation. Among the 54,103 detected cells, 17,420 cells were classified as T cell or innate lymphoid cells, which represented the most prevalent cell types. Re-

clustering revealed 12 subtypes of T/ILCs. Naive $CD4^+$ T cells (n = 1,689) showed high expression of naïve marker genes, such as *SELL and CCR7*[2] (Supplementary Fig. 2a and 2c). Effector $CD4^+$ T cells (n = 1,352) expressed the canonical Th17 cytokine genes, *IL17A* (Supplementary Fig. 2a and 2c). Memory $CD4^+$ T cells (n = 3,314) were characterized by memory marker *CCR6*[3] (Supplementary Fig. 2a and 2c). Effector $CD8^+$ T cells (n = 1,750) showed expression of key cytokines involved in tumor killing, such as *IFNG*[4] (Supplementary Fig. 2a and 2c). Exhausted $CD8^+$ cells (n = 2,040) expressed checkpoint marker genes *TIGIT* and *PDCD1* (Supplementary Fig. 2a and 2c), identity of exhausted T cells[5]. On the other hand, regulatory T cells (Treg; n = 2,319) showed typical expression for transcription factor *FOXP3* and follicular helper T cells (TFH; n = 888) for expression of B cell chemoattractant *CXCL13*[6] (Supplementary Fig. 2d and 2f). Mucosal-associated invariant T cells (MAIT; n = 508) were also identified by unique expression of *SLC4A10*[7] (Supplementary Fig. 2d and 2f), while gamma delta T cells (gdT; n = 2,058) were featured by T-cell receptor delta chain gene, *TRDC* (Supplementary Fig. 2a and 2c). Two cell types that showed great similarities to T cells, were identified, including conventional NK cells (n = 310) with expression of *FGFBP2*, and innate lymphoid cells (n = 499) with high expression of markers *KIT* and *IL23R* (Supplementary Fig. 2a and 2f)[8]. Subcluster-specific genes are listed in Supplementary Data 2. The characterization of cells from all clusters in both normal and cancerous tissues revealed in all five patients a significant increase in the infiltration of regulatory T cell infiltration (Difference [Diff]= 12.8%, p = 0.0036; paired two-sided t-test; Supplementary Fig. 2d and 2e). We also observed a significant decrease of gamma delta T cells (Diff = -4.47%; p = 0.00091) and memory $CD4^+$ T cells (Diff = -23.62%; p = 0.016) (Supplementary Fig. 2d and 2f). These differences in T cell subtypes imply a dramatic remodeling of the cellular components of tumor microenvironment.

To address the controversial prognostic role of B cells in human cancer, we further classified 2,998 B cells into 7 subclusters, including *IL10*-expressing regulatory B cells (Bregs; n = 371), $CD11C^+$ B cells with CD11C encoded by *ITGAX* (n = 229), follicular B cells (FO B; n = 346) with known markers *MS4A1* and *CD19*[9], germinal center B

cells (GC B; n = 71) which is regulated by transcription factor *BCL6*[10] and enzyme *AICDA* essential for class-switch recombination and somatic hypermutation[11], *LMO2*$^+$ B cells (n = 220) characterized by *LMO2* expression in follicular lymphoma B cells[12], non-switched memory B cells (n = 868) and switched memory B cells (n = 893) according to *IGHM* and *IGHD* expression [12] (Supplementary Fig. 2d and 2g). We found a significant decrease in the infiltration of Bregs (Diff = -9.01%; p = 0.016) in the tumor compared to adjacent normal tissue, consistent with previous observation[13] (Supplementary Fig. 2d and 2g).

The annotation of endothelial cells (EC) types was based on earlier reports[14,15]. We classified 2,205 ECs into five subtypes, including arterial (*CD36*$^+$, *ACE*$^+$, n = 632), capillary (*ESM1*$^+$, *ADAMTS5*$^+$; n = 715), lymphatic (*LYVE1*$^+$, *PROX1*$^+$, n = 219), venous (*CLU*$^+$, *ACKR1*$^+$; n = 566), and proliferating (*MKI67*$^+$; n = 63) ECs (Supplementary Fig. 2a and 2c). As previously shown in many types of tumors, capillary ECs were more enriched than other EC subtypes, such as arterial or venous ECs (Supplementary Fig. 2d and 2h). Specifically, in comparison with adjacent normal tissue, capillary ECs were dramatically increased in CRC (Diff = 61.4%; $p = 4.6 \times 10^{-4}$), and proliferating ECs also showed a statistically significant increase (Diff = 5.30%; p = 0.016; (Supplementary Fig. 2d and 2h). Conversely, venous ECs (Diff = -28.9%; p = 0.0017) and arterial ECs (Diff = 28.9%; p =0.0027) were markedly decreased (Supplementary Fig. 2d and 2h). These findings suggested that aberrant vasculature was also the characteristics of CRC TME, and the re-normalization of tumor vasculature, rather than simply blocking tumor angiogenesis, might represent a novel therapeutic strategy targeting the TME.

Taken together, the difference between the tumor- and adjacent normal-tissues- specific cell types indicating potential remodeling of TME in CRC.

Supplementary Note 3 | Comparison of MSCs subtypes with previous publication

Lee *et al.* classified MSCs into myofibroblasts, pericytes, smooth muscle cells, and stromal cells from patients with CRC in Samsung Medical Center (SMC) in Korea and Katholieke Universiteit Leuven (KUL) in Belgium[13]. To explore how the cell type annotations of our scRNA-seq data correspond to the stromal cell types characterized by Lee *et al*, we integrated our dataset with Lee's dataset using the Integration in Seurat package to generate a unified two-dimensional UMAP space (Supplementary Fig. 3a), and found our *FGFR2*$^{+}$ fibroblasts and *MFAP5*$^{+}$ myofibroblasts were mostly similar to Lee's stromal 1, *ICAM1*$^{-}$ and *ICAM1*$^{+}$ telocytes were mostly similar to Lee's stromal 2, *NT5E*$^{+}$ fibroblasts and *CD24*$^{+}$ fibroblasts were mostly similar to Lee's stromal 3, *DES*$^{+}$ myofibroblasts were mostly similar to Lee's smooth muscle cells, *FAP*$^{+}$ fibroblasts were mostly similar to Lee's myofibroblasts (Supplementary Fig. 3b-c). To investigate whether our *FAP*$^{+}$ fibroblasts were myofibroblasts or not, we plot gene expression of myofibroblast markers *MYH11*, *ACTG2,* and *TAGLN* in our dataset and Lee's, we found these markers expressed in our *DES*$^{+}$ myofibroblasts and *MFAP5*$^{+}$ myofibroblasts but not express in *FAP*$^{+}$ fibroblasts (Supplementary Fig. 3d), suggesting that *FAP*$^{+}$ fibroblasts were not myofibroblasts, but an independent stromal population.

Supplementary Note 4 | Comparison of myeloid cell subtypes with previous publication

Zhang *et al.* and Lee *et al.* have classified myeloid cells into multiple subtypes[13,16], we merged their myeloid subsets into our dataset by Seurat Integration to explore the differential cell annotation. When integrating our dataset with Zhang's dataset (Supplementary Fig. 5a-c), we found our activated DC and cDC1 are mostly similar to Zhang's cDC1-BATF3, *SPP1*$^{+}$ macrophages, *THBS1*$^{+}$ macrophages, and *VCAN*$^{+}$ macrophages are similar to Zhang's TAM-SPP1, TAM-C1QC, Macro-NLRP3, respectively. *C1QC*$^{+}$ *MRC1*$^{-}$ macrophages, neutrophil, and proliferating myeloid cells are identified in our dataset, but not Zhang's dataset, whereas Macro-PLTP, Macro-IL1B, and pDC-LILRA4 were characterized in their dataset. Through UMAP visualization, each subtype in our dataset is clearly separated, Macro-PLTP and Macro-IL1B subsets only identified by Zhang's dataset are mixed into other subtypes. As they

collected the blood samples, they also defined monocyte or monocyte-like subset in their dataset. Overall, our myeloid cells are consistent with Zhang's dataset. When our dataset integrated with Lee's dataset (Supplementary Fig. 5d-f), we found Lee's cDC is similar with our cDC2 but they didn't quantify activated DC and cDC1. As we mentioned above, they didn't perform the batch effect correction on patients or two cohorts, their macrophage subsets are mixed after integration. *C1QC*$^{+}$ *MRC1*$^{-}$ macrophages were similar with anti-inflammatory in Lee's KUL cohort, *MARCO*$^{+}$ macrophages were similar with SPP1+ in Lee's KUL cohort and SPP1+B in Lee's SMC cohort. *THBS1*$^{+}$ macrophages and proliferating myeloid cells were similar with proliferating macrophages in Lee's KUL cohort. *VCAN*$^{+}$ macrophages were similar with pro-inflammatory in both Lee's KUL and SMC cohorts. Neutrophils were quantified only in our dataset, while SPP1+A cells only quantified Lee's KUL cohort. The unknown subset from SMC and KUL cohort also showed significant difference. Overall, our dataset clearly defined myeloid subsets in CRC and improved the myeloid landscape together with previous studies.

Supplementary References

1 Newman, A. M. *et al.* Determining cell type abundance and expression from bulk tissues with digital cytometry. *Nat Biotechnol* **37**, 773-782, doi:10.1038/s41587-019-0114-2 (2019).

2 Forster, R., Davalos-Misslitz, A. C. & Rot, A. CCR7 and its ligands: balancing immunity and tolerance. *Nat Rev Immunol* **8**, 362-371, doi:10.1038/nri2297 (2008).

3 Fagin, U., Pitann, S., Gross, W. L. & Lamprecht, P. Increased frequency of CCR4+and CCR6+memory T-cells including CCR7+CD45RA(med) very early memory cells in granulomatosis with polyangiitis (Wegener's). *Arthritis Res Ther* **14**, doi:ARTN R7310.1186/ar3794 (2012).

4 Zheng, C. *et al.* Landscape of Infiltrating T Cells in Liver Cancer Revealed by Single-Cell Sequencing. *Cell* **169**, 1342-1356 e1316, doi:10.1016/j.cell.2017.05.035 (2017).

5 Guillerey, C. *et al.* TIGIT immune checkpoint blockade restores CD8(+) T-cell immunity against multiple myeloma. *Blood* **132**, 1689-1694, doi:10.1182/blood-2018-01-825265 (2018).

6 Gu-Trantien, C. *et al.* CXCL13-producing TFH cells link immune suppression and adaptive memory in human breast cancer. *JCI Insight* **2**,

doi:10.1172/jci.insight.91487 (2017).
7 Parrot, T. *et al.* MAIT cell activation and dynamics associated with COVID-19 disease severity. *Sci Immunol* **5**, doi:10.1126/sciimmunol.abe1670 (2020).
8 Crinier, A. *et al.* High-Dimensional Single-Cell Analysis Identifies Organ-Specific Signatures and Conserved NK Cell Subsets in Humans and Mice. *Immunity* **49**, 971-986 e975, doi:10.1016/j.immuni.2018.09.009 (2018).
9 Zou, Z., Ha, Y., Liu, S. & Huang, B. Identification of tumor-infiltrating immune cells and microenvironment-relevant genes in nasopharyngeal carcinoma based on gene expression profiling. *Life Sci* **263**, 118620, doi:10.1016/j.lfs.2020.118620 (2020).
10 Basso, K. & Dalla-Favera, R. BCL6: master regulator of the germinal center reaction and key oncogene in B cell lymphomagenesis. *Adv Immunol* **105**, 193-210, doi:10.1016/S0065-2776(10)05007-8 (2010).
11 Teater, M. *et al.* AICDA drives epigenetic heterogeneity and accelerates germinal center-derived lymphomagenesis. *Nat Commun* **9**, 222, doi:10.1038/s41467-017-02595-w (2018).
12 Milpied, P. *et al.* Human germinal center transcriptional programs are de-synchronized in B cell lymphoma. *Nat Immunol* **19**, 1013-1024, doi:10.1038/s41590-018-0181-4 (2018).
13 Lee, H. O. *et al.* Lineage-dependent gene expression programs influence the immune landscape of colorectal cancer. *Nat Genet* **52**, 594-603, doi:10.1038/s41588-020-0636-z (2020).
14 Smillie, C. S. *et al.* Intra- and Inter-cellular Rewiring of the Human Colon during Ulcerative Colitis. *Cell* **178**, 714-730 e722, doi:10.1016/j.cell.2019.06.029 (2019).
15 Kalucka, J. *et al.* Single-Cell Transcriptome Atlas of Murine Endothelial Cells. *Cell* **180**, 764-+, doi:10.1016/j.cell.2020.01.015 (2020).
16 Zhang, L. *et al.* Single-Cell Analyses Inform Mechanisms of Myeloid-Targeted Therapies in Colon Cancer. *Cell* **181**, 442-459 e429, doi:10.1016/j.cell.2020.03.048 (2020).
