# Supplementary material for: Single-cell and spatial analysis reveal interaction of FAP+ fibroblasts and SPP1+ macrophages in colorectal cancer
Source: Nat Commun. 2022 Apr 1;13:1742. doi: 10.1038/s41467-022-29366-6 (PMC8976074; doi:10.1038/s41467-022-29366-6)
Supplement: Supplementary file 3 — Description of Additional Supplementary Files [file 41467_2022_29366_MOESM3_ESM.pdf]

Description of Additional Supplementary Files

Supplementary Data 1

Differentially expressed genes from nine major cell types, including epithelial cells, T lymphocytes, B lymphocytes and plasma cells, myeloid cells, mast cells, endothelial cells, mesenchymal stromal cells, and glial cells. A two-sided Wilcoxon signed-rank test was used to assess statistical significance.

Supplementary Data 2

Differentially expressed genes from each subtype in major cell types, including epithelial cells, T lymphocytes, B lymphocytes, myeloid cells, endothelial cells, and mesenchymal stromal cells shown in each sheet. A two-sided Wilcoxon signed-rank test was used to assess statistical significance.

Supplementary Data 3

The processed gene expression data in count level for 54103 cells in this study.

Supplementary Data 4

The metadata for 54103 cells in this study, including barcode, Patient ID, nCount, nFeature, Tissue, percentage of mitochondrial genes, cell types, and main types.
