# Supplementary material for: Single-cell and spatial analysis reveal interaction of FAP+ fibroblasts and SPP1+ macrophages in colorectal cancer
Source: Nat Commun. 2022 Apr 1;13:1742. doi: 10.1038/s41467-022-29366-6 (PMC8976074; doi:10.1038/s41467-022-29366-6)
Supplement: Supplementary file 8 — Reporting Summary [file 41467_2022_29366_MOESM8_ESM.pdf]

Corresponding author(s): Youqiong Ye, Bing Su

Last updated by author(s): Feb 17, 2021

Reporting Summary

Nature Research wishes to improve the reproducibility of the work that we publish. This form provides structure for consistency and transparency in reporting. For further information on Nature Research policies, see our Editorial Policies and the Editorial Policy Checklist.

Statistics

For all statistical analyses, confirm that the following items are present in the figure legend, table legend, main text, or Methods section.

n/a	Confirmed	
☐	☒	The exact sample size (*n*) for each experimental group/condition, given as a discrete number and unit of measurement
☐	☒	A statement on whether measurements were taken from distinct samples or whether the same sample was measured repeatedly
☐	☒	The statistical test(s) used AND whether they are one- or two-sided<br>*Only common tests should be described solely by name; describe more complex techniques in the Methods section.*
☐	☒	A description of all covariates tested
☐	☒	A description of any assumptions or corrections, such as tests of normality and adjustment for multiple comparisons
☐	☒	A full description of the statistical parameters including central tendency (e.g. means) or other basic estimates (e.g. regression coefficient) AND variation (e.g. standard deviation) or associated estimates of uncertainty (e.g. confidence intervals)
☐	☒	For null hypothesis testing, the test statistic (e.g. F, t, r) with confidence intervals, effect sizes, degrees of freedom and P value noted<br>*Give P values as exact values whenever suitable.*
☒	☐	For Bayesian analysis, information on the choice of priors and Markov chain Monte Carlo settings
☐	☒	For hierarchical and complex designs, identification of the appropriate level for tests and full reporting of outcomes
☐	☒	Estimates of effect sizes (e.g. Cohen's *d*, Pearson's *r*), indicating how they were calculated

Our web collection on statistics for biologists contains articles on many of the points above.

Software and code

Policy information about availability of computer code

Data collection	No software was used for data collection.
Data analysis	Single-cell suspensions were loaded onto a Chromium Single Cell Controller Instrument (10x Genomics) to generate single-cell gel beads in emulsions (GEMs). Illumina sequencer was used for RNA sequencing.<br>Flow cytometry data btained by BD FACSDiva software v8.0.2 and analyzed with FlowJo v.10.5.3, and statistical analysis was done by GraphPad Prism 6.<br>All sequencing data analysis was performed by R programming (R 3.6.0) and deconvolution software CIBERSORTx in the website (https://cibersortx.stanford.edu/). R codes are deposited in GitHub: https://github.com/youqiongye/CRC_scRNAseq /tree/V1.0.0.

For manuscripts utilizing custom algorithms or software that are central to the research but not yet described in published literature, software must be made available to editors and reviewers. We strongly encourage code deposition in a community repository (e.g. GitHub). See the Nature Research guidelines for submitting code & software for further information.

Data

Policy information about availability of data

All manuscripts must include a data availability statement. This statement should provide the following information, where applicable:

- Accession codes, unique identifiers, or web links for publicly available datasets
- A list of figures that have associated raw data
- A description of any restrictions on data availability

The raw data of single cell RNA-seq and spatial transcriptomics generated in this study were deposited in Genome Sequence Archive with accession ID HRA000979. Since these data are related to human genetic resources, raw data can be obtained within half year by requesting and following the guidelines for Genome

Sequence Archive for non-commercial use at https://ngdc.cncb.ac.cn/gsa-human/request/HRA000979. There are no time restrictions once access has been granted. The guidance for making a data access request of GSA for humans can be downloaded from https://ngdc.cncb.ac.cn/gsa-human/document/GSA-Human_Request_Guide_for_Users_us.pdf. The processed gene expression data is submitted as Supplementary Data 3 and the metadata is submitted as Supplementary Data 4.

Public data collection:

1) The processed CRC public scRNAseq dataset were download from Gene Expression Omnibus (GEO, https://www.ncbi.nlm.nih.gov/geo/), including GSE146771, GSE132465, and GSE144735.

2) Normalized gene expression data based on fragments per kilobase of exon model per million reads mapped (FPKM) of colon adenocarcinoma and rectum adenocarcinoma from TCGA data portal (http://gdac.broadinstitute.org/).

3) Expression datasets based on Affymetrix microarray of CRC patients were downloaded from Gene Expression Omnibus (GEO, https://www.ncbi.nlm.nih.gov/geo/), including GSE41568, GSE39582, GSE37892, GSE33113, GSE21510, GSE20916, GSE18105, GSE13294, GSE14333, GSE23878, GSE17536, and GSE17537. The data information (e.g., sample size, overall survival times, progressive free survival time) were summarized in Supplementary Table 2.

4) The value of infiltration of lymphocyte, the richness of T cell receptor, non-silent mutation rate, and neoantigen load of CRC were obtained from https://gdc.cancer.gov/about-data/publications/panimmune.

5) The public gene expression data and detailed clinical information for patients with anti-PD-L1 (antibody: atezolizumab) treatment were obtained from IMvigor210 cohort (http://research-pub.gene.com/IMvigor210CoreBiologies, (Intervention treatment of advanced urinary tract transitional cell carcinoma).

Source data are provided in this paper, FAP and SPP1 TMA staining, Fig. 1-8, and Supplementary Fig. 1-8. The remaining data are available within the Article, Supplementary Information or Source Data file.8.

Field-specific reporting

Please select the one below that is the best fit for your research. If you are not sure, read the appropriate sections before making your selection.

- [x] Life sciences
- [ ] Behavioural & social sciences
- [ ] Ecological, evolutionary & environmental sciences

For a reference copy of the document with all sections, see nature.com/documents/nr-reporting-summary-flat.pdf

Life sciences study design

All studies must disclose on these points even when the disclosure is negative.

Sample size	In our study, a total of 54,103 cell transcriptomics from 5 patients and 8,705 spots of transcriptomics from 4 patients were retained for subsequent analysis. When reporting "n-values" for our spatial data, this relates to the number of spots that the tissue section covered, which is solely determined by the shape and character of the tissue piece being investigated.<br>Three public CRC single cell RNAseq datasets, including 27,414 cells from 6 CRC Belgian patients (GSE132465), 63,689 cells from 23 CRC Korean patients (GSE146771), and 54,285 cells from 23 CRC Chinese patients (GSE146771).<br>We collected 14 public mRNA expression dataset with colorectal cancer to assess the infiltration of cell types which defined by our single cell RNAseq data. Their sample size is listed as follows: TCGA COAD/READ (n = 635); GSE39582 (n = 566); GSE14333 (n = 290); GSE17536 (n = 177); GSE13294 (n = 155); GSE41568 (n = 133); GSE41568 (n = 133); GSE37892 (n = 130); GSE21510 (n = 123); GSE20916 (n = 111); GSE18105 (n = 94); GSE33113 (n = 90); GSE17537 (n = 55); GSE23878 (n = 35). 384 cancer patients with anti-PD-L1 (antibody: atezolizumab) .<br>For flow cytometry experiments, we used 6 biological replicates in the study.<br>For immuno-fluorescent staining, 3 patients with 3 sections in each group in the study.<br>For ELISA analysis, chemerin concentration was detected in plasma of healthy donor (n = 17) and CRC patients (n = 20).
Data exclusions	We included 14 datasets with ≥ 30 samples for further analysis.
Replication	All results represent the mean±S.E.M. from three independent experiments. All the replication were successful.
Randomization	Samples were allocated to groups based on disease status (normal and tumor tissue) if applicable.
Blinding	In our study, blinding was not applicable to this study since it is exploratory in character and have no elements that might be influenced by bias from the subject or observer.

Reporting for specific materials, systems and methods

We require information from authors about some types of materials, experimental systems and methods used in many studies. Here, indicate whether each material, system or method listed is relevant to your study. If you are not sure if a list item applies to your research, read the appropriate section before selecting a response.

Materials & experimental systems

n/a	Involved in the study	
	x	Antibodies
x		Eukaryotic cell lines
x		Palaeontology and archaeology
x		Animals and other organisms
	x	Human research participants
x		Clinical data
x		Dual use research of concern

Methods

n/a	Involved in the study	
x		ChIP-seq
	x	Flow cytometry
x		MRI-based neuroimaging

Antibodies

Antibodies used

Alexa Fluor 488 anti-human CD326 (EpCAM) (clone 9C4), Biolegend, Cat#324210, dilution 1:200;
Alexa Fluor 488 anti-human CD31 Antibody (clone WM59), Biolegend, Cat#303110, dilution 1:200;
Alexa Fluor 700, anti-human CD45 Monoclonal Antibody (2D1), eBioscience, Cat#56-9459-42, dilution 1:200;
PerCP/Cyanine5.5 anti-human CD325 (N-Cadherin) (clone 8C11), Biolegend, Cat#350814, dilution 1:200;
Brilliant Violet 510 anti-human CD146 (clone P1H12), Biolegend, Cat#361022, dilution 1:200;
Brilliant Violet 785 anti-human CD90 (Thy1) (clone 5E10), Biolegend, Cat#328142, dilution 1:200;
Brilliant Violet 421 anti-human CD24 Antibody (clone ML5), Biolegend, Cat#311122, dilution 1:200;
PE/Cyanine7 anti-human CD73 (Ecto-5'-nucleotidase) (clone AD2), Biolegend, Cat#344010, dilution 1:200;
PE anti-human CD142 (clone HTF-1), eBioscience, Cat#12-1429-42, dilution 1:200;
Alexa Fluor 700 anti-human ICAM1 (clone HA58), eBioscience, Cat#56-0549-42, dilution 1:200;
BV605 anti-human CD26 (clone L272), BD OptiBuild, Cat#745244, dilution 1:200;
APC anti-human FAP (clone # 427819), RD System, Cat# FAB3715A-100, dilution 1:200;
BV711 anti-human CD3 (clone UCHT1), BD Horizon, Cat#563725, dilution 1:200;
BV786 anti-human CD19 (clone HIB19), BioLegend, Cat#302240, dilution 1:200;
BV510 anti-human CD16b (clone CLB-gran11.5)(RUO), BD OptiBuild, Cat#744968, dilution 1:200;
APC-Fire700 (APC-Cy7) anti-human XCR1 (clone S15046E), BioLegend, Cat#372608, dilution 1:200.
APC anti-human CD1C (clone L161), BioLegend, Cat#331524, dilution 1:200.
PE/Cyanine7 anti-human CD209 (clone 9E9A8), BioLegend, Cat# 330114, dilution 1:200;
Alexa Fluor® 700 anti-human CD14 (clone HCD14), BioLegend, Cat#325614, dilution 1:200;
Brilliant Violet 605™ anti-human CD45 (clone HI30), BioLegend, Cat#304042 PE anti-human CD13(clone WM15), BioLegend, Cat#301704, dilution 1:200;
PercP-Cy5.5 anti-human CD163 (clone GHI/61), BD Pharmingen, Cat#563887, dilution 1:200;
PE-TexaRed (PE-CF594) anti-human CD206 (clone 19.2), BD, Cat#564063, dilution 1:200;
Anti-Fibroblast activation protein, alpha antibody [EPR20021], Abcam, Cat# ab207178, dilution 1:50;
Goat Anti-Rabbit IgG H&L (Alexa Fluor 647) preadsorbed, Abcam, Cat# ab150087, dilution 1:50;
Anti-Osteopontin antibody [EPR 21139-316], Abcam, Cat# ab214050, dilution 1:1000.

Validation

The validation of all of the antibodies depends on product datasheet and published literature.

Human research participants

Policy information about studies involving human research participants

Population characteristics

Adjacent normal mucosa and CRC tumor tissues were collected from Ruijin Hospital Affiliated to Shanghai Jiao Tong University, Renji Hospital Affiliated to Shanghai Jiao Tong University, and The First Affiliated Hospital of University of Science and Technology of China.

Recruitment

All patients with Colorectal cancer that was planed to undergo primary surgery could be asked to be recruited. All patients provided written consent to participate in the study approval of local medical ethnics.

Ethics oversight

The study protocol was approved by the Institutional Review Board of Ruijin Hospital Affiliated to Shanghai Jiao Tong University, Renji Hospital Affiliated to Shanghai Jiao Tong University, and The First Affiliated Hospital of University of Science and Technology of China.

Note that full information on the approval of the study protocol must also be provided in the manuscript.

Flow Cytometry

Plots

Confirm that:

- [x] The axis labels state the marker and fluorochrome used (e.g. CD4-FITC).
- [x] The axis scales are clearly visible. Include numbers along axes only for bottom left plot of group (a 'group' is an analysis of identical markers).
- [x] All plots are contour plots with outliers or pseudocolor plots.
- [x] A numerical value for number of cells or percentage (with statistics) is provided.

Methodology

Sample preparation	Fat tissue and visible blood vessels were removed before tissue process. Fresh normal mucosa and CRC tissue were washed with ice-cold PBS, and cut into small pieces. For normal mucosa, tissues were placed and shaken into 10 mL of EDTA-containing buffer (5 mM EDTA, 15 mM HEPES, 1 mM DTT, and 10% FBS-supplemented PBS) for 1 hour at 37 °C. Tumor tissues were incubated with 10 mL of DTT (65 mM)-containing PBS (supplemented with 10% FBS) for 15 min at 37 °C with shaking. EDTA and DTT was removed after above incubation with PBS twice. Small tissue pieces were minced and digested with collagenase VIII at 0.38 mg/mL and DNase I at 0.1 mg/mL in complete RPMI1640 medium (containing 10% FBS, 100 U/mL penicillin and 100 mg/mL streptomycin) for 1 hour at 37 °C. After digestion, tubes were shaken vigorously for 5 min. 21-gauge syringes were used to dissociate cells mechanically. Cells were filtered through 100 µm filter and pelleted and washed with PBS twice. Freshly prepared cell suspensions were ready for scRNA-seq and flow cytometry staining.
Instrument	FACS analysis was performed on BD Symphony.
Software	Data was acquired using BD FACSDiva software v8.0.2, and analyzed using FlowJo 10.5.3; Statistical analysis was done by two-sided paired t-test with GraphPad Prism 6
Cell population abundance	We performed FACS analysis but didn't sorting. The abundance is not available.
Gating strategy	The gating strategy for glial cells (live CD45- EPCAM- CD45- CDH2+ CD146+), pericytes and myofibroblasts (live CD45- EPCAM- CD45- CDH2- CD146+), telocytes (live CD45- EPCAM- CD45- CDH2- CD146- CD142+), total fibroblasts (live CD45- EPCAM- CD45- CDH2- CD146- CD142- CD90+), CD24+ fibroblasts (live CD45- EPCAM- CD45- CDH2- CD146- CD142- CD90+ CD26- CD24+), CD26+ fibroblasts (live CD45- EPCAM- CD45- CDH2- CD146- CD142- CD90+ CD24- CD26+), NT5E+ fibroblasts (live CD45- EPCAM- CD45- CDH2- CD146- CD142- CD90+ CD24- CD26- CD73+) and FAP+ fibroblasts (live CD45- EPCAM- CD45- CDH2- CD146- CD142- CD90+ CD24- CD26- CD73- FAP+). Telocytes were further gated based on ICAM1 expression.<br>The gating strategy of myeloid subtypes in human CRC tissue. Macrophages were gated by exclusion the expression of CD3 (T cell marker), CD19 (B cell marker), XCR1, CD1C (DC1 and DC2 respectively), and CD16B (neutrophils marker), and were CD45 positive and CD14 positive. SPP1+ macrophages were both CD206 and CD13 positive. THBS1+ macrophages were CD13 negative and CD209, CD206 positive.

- [x] Tick this box to confirm that a figure exemplifying the gating strategy is provided in the Supplementary Information.
